# Human resource governance: what does governance mean for the health workforce in low- and middle-income countries?

**DOI:** 10.1186/1478-4491-11-6

**Published:** 2013-02-15

**Authors:** Avril D Kaplan, Sarah Dominis, John GH Palen, Estelle E Quain

**Affiliations:** 1 Abt Associates Inc, 4550 Montgomery Ave, Bethesda, MD 20814, USA; 2 United States Agency for International Development, 1300 Pennsylvania Ave NW, RRB 5.10.74, Washington, DC 20523, USA

**Keywords:** Health governance, Health workforce, Human resources for health, Health system strengthening, Human resource management

## Abstract

**Background:**

Research on practical and effective governance of the health workforce is limited. This paper examines health system strengthening as it occurs in the intersection between the health workforce and governance by presenting a framework to examine health workforce issues related to eight governance principles: strategic vision, accountability, transparency, information, efficiency, equity/fairness, responsiveness and citizen voice and participation.

**Methods:**

This study builds off of a literature review that informed the development of a framework that describes linkages and assigns indicators between governance and the health workforce. A qualitative analysis of Health System Assessment (HSA) data, a rapid indicator-based methodology that determines the key strengths and weaknesses of a health system using a set of internationally recognized indicators, was completed to determine how 20 low- and middle-income countries are operationalizing health governance to improve health workforce performance.

**Results/discussion:**

The 20 countries assessed showed mixed progress in implementing the eight governance principles. Strengths highlighted include increasing the transparency of financial flows from sources to providers by implementing and institutionalizing the National Health Accounts methodology; increasing responsiveness to population health needs by training new cadres of health workers to address shortages and deliver care to remote and rural populations; having structures in place to register and provide licensure to medical professionals upon entry into the public sector; and implementing pilot programs that apply financial and non-financial incentives as a means to increase efficiency. Common weaknesses emerging in the HSAs include difficulties with developing, implementing and evaluating health workforce policies that outline a strategic vision for the health workforce; implementing continuous licensure and regulation systems to hold health workers accountable after they enter the workforce; and making use of health information systems to acquire data from providers and deliver it to policymakers.

**Conclusions:**

The breadth of challenges facing the health workforce requires strengthening health governance as well as human resource systems in order to effect change in the health system. Further research into the effectiveness of specific interventions that enhance the link between the health workforce and governance are warranted to determine approaches to strengthening the health system.

## Background

A growing understanding of how the different parts of a health system interact is highlighting how human resource constraints are impeding system strengthening in low- and middle-income countries (LMIC). As early as 2004, the Task Force on Health Systems Research identified human resources for health (HRH) as two of 12 health systems research priorities for achieving the Millennium Development Goals [1]. In a 2010 study, Ranson et al. used a comprehensive literature review, key informant interviews, and a consultative multinational workshop to prioritize HRH research questions that LMIC countries should answer when attempting to strengthen their health workforce [2]. The study produced 21 research questions on training; regulatory, financial and organization mechanisms; and planning, policy development, and inter-sectoral collaboration. These questions reflect the fact that the available literature is mainly descriptive, looking at individual country initiatives to improve HRH recruitment, retention, and distribution.

While this literature has been met by action-oriented initiatives such as the Global Health Workforce Alliance and Frontline Health Workers Coalition, which are increasing awareness of HRH issues through communication and education campaigns, there has been little examination of the structural issues that limit the effectiveness of the health workforce and indeed of health systems. This examination is driven by two main weaknesses. First, strong health systems require integrated solutions, not just narrowly focused interventions. Chee et al. argue that a health system is strengthened through comprehensive changes to performance drivers, such as the relationships across health system functions, as opposed to improving individual support aspects of the system [3]. Similarly, a 2009 World Health Organization (WHO) paper affirmed that it is “the multiple relationships and interactions among the [health system] building blocks—how one affects and influences the others, and is in turn affected by them—that converts these blocks into a system” [4].

Second, in examining the health workforce interaction with the larger health system, we need to consider the strong link between health workforce development and health governance. In the World Health Report 2000, the WHO introduced the concept of stewardship as one of four health system functions [5]. The WHO 2007 report *Everybody’s Business: Strengthening Health Systems to Improve Health Outcomes* classifies leadership and governance as one of six health system building blocks, and the health workforce as a second building block [6]. However, the concept of governance is rarely examined in the HRH literature.

Attention to the link between health workforce development and governance is growing, albeit not quickly enough. A 2010 conference on “Responsible Governance for Improved Human Resources for Health,” organized by the Royal Tropical Institute in Amsterdam, attracted 181 participants from 31 countries [7]. Still, the authors of this paper found only one review of the relationship between governance and the health workforce [8]. The conference concluded that further conceptualization, evidence building, and documentation of successful HRH strengthening strategies is needed [8].

All these observations point to the need to continue to refine systematic and integrated approaches to health system strengthening, as well as to bridge the gap between health workforce and governance, in order to maximize the effectiveness of service delivery. This paper focuses on the latter topic.

## Methods

The authors used a three-pronged methodology: 1) a literature review to identify the key principles of governance; 2) development of a framework based on the literature review that assigns indicators and describes linkages between governance and the health workforce and 3) an analysis of country Health System Assessment (HSA) data to determine how countries are operationalizing health governance to improve health workforce performance.

### Literature review findings

A literature review was conducted to identify key principles of health governance. Online search engines such as BioMed, PubMed and Google Scholar were used to identify documents using keywords such as “health governance,” “stewardship,” “leadership and management” and “health governance indicators.” A total of 43 reports and articles were determined to be relevant for health governance dating back to 1999.

In the health literature, ‘governance’ has several definitions and theoretical frameworks. Several authors have defined health stewardship [9,10] and health governance [11-14] over the past decade. The WHO definition of leadership and governance is the existence of a strategic policy framework as well as effective oversight, coalition building, regulation, attention to system design, and accountability [6]. Based on the literature review, the authors of this paper define health governance as the set of rules that define the responsibilities of health system actors, how they operate, and how they relate to one another.

The review of health governance literature identified eight health governance principles. They provide an outline for the rest of this paper.

1. Strategic vision and direction [5,13,15,16]: A strategic vision and direction helps to define priorities and expected roles of health system actors, establish benchmarks for measuring short- and medium-term performance, and build consensus among different stakeholder groups to align their programs with government priorities [5].

2. Accountability [5,13,15-20]: Accountability involves “holding public officials and service providers answerable for processes and outcomes and imposing sanctions if specified outputs and outcomes are not delivered” [17]. Accountability applies to multiple health system actors, including policymakers, planners, managers, providers, and support workers.

3. Transparency [13,15,17-19]: Transparency refers to openness and clarity in decision-making and allocation of resources [13,18]. Countries require systems for transparent decision-making, budgeting, and tracking of expenditures.

4. Information generation [5,13,16,17,21]: Timely, accurate information enables stakeholders to make evidence-informed policies, and to take action when goals and standards are not met [17]. Information is relevant to decision makers throughout the health system, at the policy, program, and management levels.

5. Efficiency [18,19,22]: Efficiency refers to the “extent to which limited human and financial resources are applied without unnecessary waste, delay, or corruption” [18].

6. Equity and fairness [13,15,18,21]: Equity and fairness relate to the degree in which policies and procedures apply equally to everyone [18].

7. Responsiveness [10,13]: Responsiveness is the ability of the government and other institutions to respond to population health needs at both the regional and local levels [13].

8. Citizen voice and participation [13,15,18,19]: Voice and participation involve individuals acting through institutions that represent their interests, and the interests of a larger group [15].

### Development of a health workforce and health governance framework

Multiple resources and tools are available to assess health governance performance [23-28] and health workforce performance [25-32]. However, none were identified that assess the overlap between the two.

To address the gap in assessment tools, the authors used the results of the governance literature review and indicators from an existing tool, the Health System Assessment (HSA), to develop a framework that demonstrates the linkage between the health workforce and health governance. The HSA is an indicator-based assessment described in the *Health System Assessment Approach: A How-to Manual* and developed by the Health Systems 20/20 project with support from the United States Agency for International Development [33]. The HSA analyses a country’s health system performance according to a detailed set of internationally recognized qualitative and quantitative indicators. The HSA incorporates indicators across all six of the WHO health system building blocks^a^ to assess public and private sector performance. The assessment process includes a review of available literature and statistics, key informant interviews, and field visits. Teams applying the HSA approach are encouraged to identify cross cutting themes across the six building blocks, and the HSA Assessment Approach manual has been vetted and updated over time to delineate linkages. This approach has been applied in more than 20 countries between 2007 and 2012, allowing for cross country themes to emerge that portray strengths, weaknesses and areas for further research.

The HSA devotes a chapter to the HRH building block. The HRH chapter uses a variety of health workforce indicators which are organized according to the widely recognized HRH Action Framework (HAF). The HAF was developed through a series of expert consultations led by the Global Health Workforce Alliance and bilateral and multilateral agencies [34]. The HAF aims to create an enabling environment for effective human resource management by strengthening six “action fields” that include: policy, finance, education, partnerships, leadership and human resource management. According to the HAF, implementing country-specific interventions within each of these action fields will lead to an improved health workforce that is able to deliver more effective, efficient, equitable and higher quality services, and thereby improve population health outcomes [34].

The authors identified a number of cross-cutting themes among the eight governance principles and six HAF action fields. These cross-cutting themes were used to develop a Health Workforce and Governance Framework that uses HSA indicators to highlight the relationship between HRH and governance (Table 1). The Framework guides this analysis of the results of 20 HSAs to determine best practices and common challenges in the intersection of governance and HRH in low- and middle-income countries.^b^

**Table 1:** **Linkages between health workforce and governance**^**a**^

**Governance principle**	**Health workforce/governance link**	**HSA**^**b **^**indicator**	**HAF**^**c **^**action field**
**Information**	Information systems facilitate production of data to inform decisions about planning/training/supporting the health workforce.	Availability/use of HRH^d ^information systems	**Human resource management systems**
	Bottom up information from health workers assists government to: formulate evidence-based policy, plan direction of health sector, and monitor performance.	Public/private sector providers report information to government	
**Accountability**	Environment where health workers know responsibilities and have supportive supervision, and supervision enables them to better fulfill duties.	Enabling environment exists to achieve goals and targets	
	Existence/use of tools to measure health worker performance enables managers to hold workers accountable to set expectations.	Availability of mechanisms to monitor and improve performance	
	Scopes of practice (i.e., registration, licensure) ensure qualifications are met upon entry into profession and reassessment procedures are in place to ensure staff maintains qualified status.	Existence of clear and up-to-date scopes of practice	**Policy**
**Strategic vision**	Evidence-based and costed HRH policies/strategic plans provide a vision for the health workforce and help to coordinate activities within the health sector.	Existence and use of up-to-date HRH policies/strategic plan	
**Transparency**	Documentation ensures clarity among health workers concerning the rules they are governed by.	Employment policies documented/used	
	Routine NHA^e ^data enable stakeholders to track health expenditures from sources to providers.	NHA reports expenditure data	**Finance**
	Transparent/comprehensive account of the budget process ensures clarity in decision-making.	Budgets/projections done for HRH	
**Efficiency**	If implemented appropriately, financial and non-financial incentives can ensure better performance with less waste.	Services organized/financed to incentivize providers to improve care	
	Performance contracting, whereby public sector collaborates/purchases services from private sector, can lead to delivery of better quality care at a lower cost.	Contracting mechanisms exist between MOH^f^/public/private providers	
	Informal user fees act as a barrier to care and increase costs without improving quality or access to public health services.	Existence of informal user fees in the public sector	
	Mechanisms used to pay health service providers serve as an incentives/affect the quality of care.	Type of provider payment mechanisms	
**Equity**	Perceptions of unfair wages and actual wage differences drive staff turnover. Salaries should be equitable among employees completing similar levels of work, and paid on time.	Salaries competitive in local/regional labor markets and paid on time	
	Providers recruited from and then posted to rural areas are more likely to stay in rural areas.	Urban versus rural admissions/graduates	**Education**
**Responsiveness**	Aligning pre-service education with the competencies needed to address population health enables the right numbers and cadres to enter the workforce with the right skills.	Production of new health care workers responsive to population health needs	
	Outdated curriculum is unresponsive to population health needs and a source of poorly trained workers.	Pre service education regularly updated	
	In-service training should be linked to organizations’ priorities/changes in the health sector. Ad-hoc in-service training that is unrelated to staff needs often results in low attendance rates.	In-service training aligned with population/workforce needs	
	High-level government officials (ministers, parliament, cabinet members, private health sector leaders) should be aware of HRH issues to develop calls for action/include HRH in donor requests.	Awareness of high-level government officials of HRH issues	**Leadership**
**Voice and participation**	Communities should have a voice to determine which services are provided/how funding is budgeted/provide feedback on service quality.	Mechanisms in place for patient and community feedback	**Partnerships**

^a^Adapted from the *Health System Assessment Approach: A How-to Manual.*

^b^Health System Assessment.

^c^Human Resources for Health Action Framework.

^d^Human Resources for Health.

^e^National Health Accounts.

^f^Ministry of Health.

### Analysis of country data

To gain a better understanding of how countries are applying the eight principles of governance to improve health workforce performance, a team of analysts used QSR NVivo Software 9 to analyze data from HSA reports completed between 2007 and 2012 (QSR International Pty Ltd, Doncaster, Victoria, Australia). The countries included in this analysis are presented in Table 2. The authors developed a code book of organizational nodes based upon the Health Workforce and Governance Framework, and organized the nodes according to the eight governance principles. All HRH data produced by the HSAs were analyzed according to these nodes, and major themes were identified. The research team gathered best practices and common challenges from within each theme to illustrate the practical application of governance principles to improve health workforce performance.

**Table 2:** Health systems assessments, by country and year

**Country**	**Year**
Angola	2010
Antigua and Barbuda	2011
Cote d’Ivoire	2010
Dominica	2012
Grenada	2011
Guyana	2010
Kenya	2010
Lesotho	2010
Mozambique	2012
Nigeria	2009
Senegal	2009
St. Vincent and Grenadines	2012
Southern Sudan^a^	2007
St. Lucia	2011
St. Kitts and Nevis	2012
Tanzania	2010
Uganda	2011
Ukraine	2011
Vietnam (two provinces)	2009
Zimbabwe	2010

^a^The HSA was completed in southern Sudan before statehood was gained.

## Results

### Strategic vision and direction

We examined the findings of 20 HSAs to determine whether the countries had an HRH policy or strategic plan outlining goals and priorities for their health workforce. We explored the existence, contents, implementation, and monitoring and evaluation of the policy or strategic plan to determine the challenges common to the countries.

#### Development and contents of HRH policy/strategic plan

The HSAs in about half of the countries indicate that HRH strategic plans and a rationalized process for health workforce training, recruitment, and deployment are in place. The plans in Uganda and other countries included specific goals and interventions for the scale-up of health workers with an appropriate skill mix, improved health resource management for planning, improved quality of in-service training, development of incentive programs, and strengthening of accreditation and licensure.

The other half of the countries examined did not have HRH-specific strategic plans in place. Antigua and Barbuda, Dominica, and southern Sudan cited constraints such as the lack of an office of strategic planning, or lack of staff to develop a plan. Absence of a strategic plan led to problems such as difficulties in managing staffing patterns, which in turn resulted in staffing shortages and an inefficient use of resources, as well as inability to coordinate with other health system priorities.

#### Implementation

Virtually every HSA reported challenges in strategic plan implementation due to limited financial and human capacity; weak endorsement of the vision at the leadership level; and poor coordination between the public and private sectors and donors.

Some strategic goals that were achieved turned out to be less effective than anticipated, because larger system weaknesses were not simultaneously addressed. For example, Lesotho’s Human Resources Development and Strategic Plan (2005–2025) contains targets for HRH production, training, and management. While the country successfully ramped up health worker training, the new health workers have not been sufficiently absorbed into the workforce because the Ministry of Health (MOH) and the Public Service Commission had not reached agreement on creating new positions.

#### Monitoring and evaluation

The HSAs indicate that nearly every country with an HRH strategic plan failed to put in place a corresponding monitoring and evaluation plan to track progress toward the stated goals. As a result, MOH staff had limited awareness of implementation progress and challenges. While some countries recognized the need to establish monitoring and evaluation indicators to identify and remedy weaknesses encountered during plan implementation, such as the percentage of cadres being absorbed into the civil service, they instead focused on broad health system outputs, like the percentage of newly trained workers.

### Accountability

We looked at various measures countries commonly use to hold health workers accountable to deliver high-quality services. These include the enabling environment in which health workers operate, mechanisms to improve performance and productivity, and existence of up-to-date scopes of practice.

#### Enabling environment

To assess the enabling environment, the HSA measures the existence and application of job descriptions and the supervision of health workers. In Kenya, Nigeria, St. Lucia, St. Vincent and the Grenadines, and Ninh Binh and Can Tho provinces in Vietnam, job descriptions are updated regularly and appear to be well understood by health workers. In Grenada, Guyana, and Lesotho, systems are in place to subject job descriptions to periodic review, but this is often not done. Outdated job descriptions reportedly led to staff being unclear about roles and responsibilities, reporting structures, and oversight responsibilities. Additionally, they impeded hiring of the right person for the position. In Guyana, health care workers’ job descriptions are reviewed biannually through informal discussions among workers and supervisors, but job descriptions for some MOH and regional personnel have not been reviewed for 20 years. Overall in the countries examined, the HSAs report that it is common to find staff performing duties that are outside of their scope of work in order to accommodate immediate circumstances.

Nine HSAs (Angola, Antigua and Barbuda, Grenada, Guyana, Kenya, Nigeria, Uganda, Vietnam, and Zimbabwe) reported a lack of capacity and resources to adequately supervise health care providers. Several interventions have been considered to remedy the situation. One is to improve supervision through mentorship programs. In Antigua and Barbuda, experienced physicians mentor newly trained physicians practicing in the same public sector facility throughout their first year of service in order to provide close technical supportive supervision and address any gaps in training. In Angola, the government often contracts foreign doctors for short periods of time to fill the gap in physicians; it is considering including ability to mentor as a hiring criterion and a formal part of the doctors’ scope of work. Countries are also taking steps to standardize supportive supervision across the health system. Angola is currently pushing to integrate supervision across vertical donor programs, and Kenya uses a standard checklist that is specific to each level of service delivery. Nigeria is integrating administrative and clinical supervision to create efficiencies that will allow a greater number of visits to be conducted; in some states, the rate of supervision visits planned and accomplished was as high as 90 percent.

#### Mechanisms to monitor and improve performance

In four countries (Guyana, Kenya, Nigeria, and Ukraine), the HSAs indicate that governments require performance reviews to hold providers accountable, but the reviews often are not carried out. In Guyana, performance reviews are to be completed annually by supervisors at the regional level using rules set by the Ministry of Public Service. In practice, reviews take place at the initiative and discretion of supervisors. In addition, there is no evidence that performance reviews inform personnel decisions such as promotions, raises, or non-pay benefits. In Kenya, ministry guidelines indicate that a performance review is to take place once per year with the goal of evaluating the previous year’s performance, planning the future year, and discussing training that could improve the worker’s performance. However, HSA interviewers could not find any providers who had received a written evaluation in the preceding five years. Dominica, in contrast, has been largely successful in implementing their annual Employee Assessment and Development Review, which is based on key result areas and performance objectives. Health workers’ annual salary increases are dependent on this process. There is positive feedback on the use of the tool, although managers reported that the link to salary increases and job stability could be stronger.

#### Clear and up-to-date scopes of practice

Nearly every country assessed requires registration of health workers in the public sector. As a tool for monitoring the active workforce, however, registration systems are limited. In Lesotho, providers register only once; no updates are required. Dominica, Kenya, and Angola require only physicians to re-register on a ‘regular’ basis. The HSAs report that a lack of registration updates makes it difficult to stay current with providers; officials do not know whether health workers are still practicing or even if they are in the country and therefore they cannot assess current health care shortages and surpluses.

Most of the HSAs indicate that countries have licensure procedures in place. Some countries require an examination before issuing a license, while others automatically license providers after graduation, for a fee. Licensure procedures are most stringent for physicians; other health cadres are less likely to have active licensure programs. Angola, Nigeria, and others do not license all health professionals. For example, Angola does not have licensure bodies for nursing and pharmacy and does not require licensure of allied health professionals, physiotherapists, and opticians. In addition, as the private sector grows in LMIC, licensure requirements for private providers have not kept up.

Due to countries’ resource limitations, the HSAs indicate that licensure is usually a one-time process. While countries increasingly require continuing education to maintain licensure, enforcing such requirements is resource-intensive.

### Transparency

We assessed countries’ adherence to the governance principle of transparency in the context of the country’s financing, and in the employment policies documented and used.

#### Budget development

The sheer magnitude of the HRH budget allocation speaks to the importance of transparency within the budgeting process. For example, 71 percent of the total health sector budget is allocated to salaries in Kenya, while 17 percent is allocated to salaries in Benin. In Lesotho, Cote d’Ivoire, and Uganda, wages represent significant proportions of recurrent costs, at 34, 40, and 53 percent respectively at the time of the HSA.

HRH budgets in the countries assessed followed two distinct budgeting processes: development at the central MOH, resulting in budgets that have little input from local levels and facilities, or development at lower levels, with budget requests flowing from districts to regions, provinces, or states and then to the central MOH. In Uganda, where the HRH budget is developed at the central level, districts are required to conform to floor and ceiling guidelines. Similarly, in southern Sudan, central-level budgets mean that states are unable to adequately assess and forecast their needs. Kenya employs a mixed approach: the budget for HRH remains under central control, but salaries are funded separately from operating expenses under the control of community health committees.

#### Availability of HRH financial data

National Health Account (NHA) estimations have been completed in fourteen of the countries examined: Angola, Cote d’Ivoire, Benin, Kenya, Mozambique, Nigeria, Senegal, southern Sudan, St. Vincent and the Grenadines, Tanzania, Ukraine, Uganda, Vietnam and Zimbabwe. However, the HSAs reveal that in many of these countries, the public does not have access to the NHA reports. In other countries, HRH financial data are not kept current.

#### Employment policies

Provider salaries generally do not take into consideration the different factors that influence the cost of living in a specific region, or the factors that motivate strong performance. Instead, countries such as Kenya, Lesotho, Nigeria, and Vietnam use salary scales that adjust for level and tenure. In contrast, in Uganda, wages are allocated to providers based on level of care they provide, their specialty, and the number of health workers in a given district, facility, or institution. In general, the HSAs indicate that salary scales are not automatically made available to the public, nor are they easy to obtain; an exception is Vietnam, where the national rule for compensation and benefits has been widely available since 2002.

Many countries, including Lesotho, southern Sudan, and St. Lucia, were found to have clearly defined and transparent career paths for some health workers, but not for all, such as nurses and community health workers. For example, in St. Lucia, 68 percent of the community health nursing cadre works on a seasonal basis or on month-to-month contracts. Many countries rely extensively on community health workers to provide essential services in rural and remote areas, but do not have measures in place to adequately compensate them or promote their careers.

### Information generation

We explored countries’ ability to generate and use information specific to HRH.

#### Availability and use of human resource information systems

In some countries, the HSAs report that data collection remains primarily paper based, resulting in issues with data quality and inefficiencies. In St. Kitts and Nevis, the paper-based information system presents challenges in collecting timely information on the existing public health workforce, monitoring wage structures and benefits, assessing geographical distribution by employment, and reviewing required educational profiles and competencies for health workforce needs.

In contrast, Ukraine has made progress in automating its human resources information system to increase the efficiency of collecting information. The system is managed by the Center for Medical Statistics, housed in the MOH, and responsibility for data collection is decentralized through the system, with clearly defined roles for districts and regional health administrations. All data reported up to the MOH are submitted on standardized forms. As a result of a clearly defined and standardized system, Ukraine has a culture of regular data collection and reporting and many components of the HIS are institutionalized.

#### Provider reporting

Public and private providers are responsible for reporting information on key health statistics. Ensuring that private providers report data was cited as a challenge in several HSAs, such as Angola, Nigeria, and Vietnam. Countries with automated information systems, such as Angola and Lesotho, have found it important to increase coordination in order to reduce the reporting burden on providers.

#### Use of information by patients and providers

While some countries, such as Angola and Guyana, have been successful in increasing access to data, most HSAs report that use of health data on behalf of patients and providers is still a challenge. Four key challenges identified in the Lesotho, Nigeria, and Vietnam HSAs impede the use of data: limited capacity to analyze data, limited feedback given to the provider level after data collection has been completed, ad hoc demand for data, and nonsystematic use of data. On the other hand, in Grenada, use of data has become institutionalized at the provider level. Community and district-level nurses receive training on results-based planning, and health centers are required to prepare annual work plans with activities linked to indicators. During facility management meetings, progress is compared to targets and service delivery and surveillance reports are reviewed.

### Efficiency

We examined the existence of informal user fees in each country and the mechanisms used to pay providers. In addition, we examined the use of incentives and public-private partnerships (PPPs) to deliver more efficient and effective services.

#### Informal user fees

The HSAs for Kenya, southern Sudan, Uganda, and Ukraine all reported that informal user fees were high and a barrier to accessing care. However, only the HSAs in Kenya and Ukraine quantified the magnitude of under-the-table payments made to providers. In Kenya, a 2007 household expenditure survey estimated that KSH 7 billion was paid to providers, whereas a utilization survey estimated expenditures of KSH 1.5 billion over the same period. In Ukraine, the 2005 NHA estimated that informal payments accounted for 10 percent of total health expenditure and 22 percent of total household expenditures. Vietnam was the only HSA that explicitly reported that informal user fees were not a barrier to accessing care.

#### Provider payment mechanisms

In Angola, Ukraine, and Vietnam, the HSAs indicate that governments pay providers on a fee-for-service basis. In Guyana, Grenada, and St. Kitts and Nevis, the government pays public provider salaries, and the private sector is paid on a fee-for-service basis. Tanzania has a unique system in that the National Health Insurance Fund (NHIF) reimburses providers on a fee-for-service basis, whereas Social Health Insurance Benefits (SHIB) use capitation to reimburse providers. The HSA reports that private providers prefer the SHIB payment method to that of the NHIF.

#### Incentives

To adjust for situations in which pay is inequitable and unfair or where working conditions are undesirable, countries are implementing a variety of innovative initiatives. The Kenya and Lesotho HSAs reported using pilot pay-for-performance mechanisms to rate and reward health centers and to promote greater productivity of health workers. Ministry of Health and Social Welfare employees in Lesotho receive two increments in their salaries annually, one of which is awarded on the yearly anniversary of service, and the other which is awarded to all civil servants at the annual budget reading. Health workers in almost all countries reported receiving supplemental income from donor projects. Mozambique has provided subsidized mortgages and homes in areas where there are health worker shortages. In addition, subsidies to work in rural areas are provided by the governments of Angola, Kenya, Lesotho, and Mozambique. Non-financial employee recognition programs are used in Mozambique by posting a photograph of the best worker of the month, and providing honors, awards, and public praise to high achievers.

#### Contracting mechanisms between government and private providers

The HSAs indicated that PPPs, in which a degree of commercial and financial risk is transferred to the private sector for the provision of a public service, are rare. The Lesotho HSA provides one of the only examples: the country’s largest hospital, the Queen Elizabeth II, is partially financed and operated by the Tsepong consortium through an 18-year PPP agreement. Tanzania has promoted PPPs in its Health Sector Strategic Plan III, and Uganda is developing its first PPP policy.

### Equity/fairness

We assessed equity and fairness in relation to the compensation of health workers and the ratio of rural to urban students admitted and graduating from training programs.

#### Adequacy and competitiveness of compensation

The HSAs indicated that adequacy of pay has implications on the morale of workers, their performance, and their decision to migrate to other areas within or outside of the country. In several countries, inadequate compensation was cited as a primary reason for migration. This should force countries to consider equity of pay not only within their countries, but also in comparison to neighboring countries. In Angola and Cote d’Ivoire, public health workers reportedly earn good salaries in comparison to other public servants and neighboring countries, whereas in Benin, salaries were lower than in neighboring countries. The HSAs indicate that countries were mixed in adjusting for inflation; some adjusted annually while others had not given increases in several years, and as a consequence had salaries that were low as compared to others in the region.

#### Ratio of urban to rural admissions and graduates

Every country examined had disparities in the deployment of rural versus urban health workers, which impacts the equitable distribution of health services throughout a country. However, only the Guyana HSA provided information on prioritizing rural students in training institutions as a mechanism to reduce geographic imbalances. Despite this policy, most students admitted to and graduating from health training were from urban regions, likely a result of the poorer educational opportunities that rural students face throughout their lifetime.

### Responsiveness

We examined whether the production of health workers reflects the health needs of the population, whether pre- and in-service training institutions equip health workers with the skills they need to address to community needs, and whether high-level government officials are aware of HRH issues and prioritize HRH interventions in response.

#### Production of health workers

Nearly every HSA examined reveals that countries lack the health workers required to address the needs of their population. In response, countries are opening new medical schools, training new cadres of health workers, and importing physicians as a short-term remedy. For example, Angola opened five new medical schools, has reintroduced community health workers to provide home visits and community education, and has imported doctors when necessary. Guyana has also imported doctors to address a critical shortage—as recently as 2009, 96 percent of physicians were expatriates. While this helps fill the immediate gap, it raises questions of sustainability of the health workforce in the long term and introduces the problems of language and cultural differences between patients and providers.

The key constraints reported in the HSAs to increasing the number of health workers have been a lack of instructors, high tuition fees that inhibit entry into training institutions, and retirement of specialists or general practitioners who cannot be replaced at the rate in which they are leaving service. In Uganda, the government developed a policy in 2000 to privatize higher education. As a result, more training institutions have been established, but most tuition fees are paid out of pocket making training programs less accessible. Nearly all HSAs reported that countries have critical shortages among physicians, pharmacists, mental health specialists, and environmental specialists. In Zimbabwe, the health workforce is staffed to only 57 percent of capacity, and in southern Sudan, 40 percent of health workers have less than one year or no training.

#### Responsiveness of pre- and in-service education

The Kenya, Uganda, and Ukraine HSAs reported that there was a lack of pre-service training for management and pharmacy. The Guyana, Kenya, Lesotho, and Vietnam HSAs indicated that their in-service training is overall uncoordinated, and dependent on non-governmental organization (NGO) involvement. In addition, when in-service training is not mandatory, it is unlikely to be completed.

Tanzania, Ukraine, and St. Vincent and the Grenadines have made progress in improving their in-service training. The government of Tanzania introduced a policy that encouraged practicing health workers to improve their skills by receiving additional training while maintaining their salary and post. The MOH and worker share the cost of training. In Ukraine, the ministry initiated a points-based system for continuing medical education of physicians, whereas in St. Vincent, trainings are held every Wednesday for nurses in order to improve skills that range from clinical best practices to management.

#### Commitment of high-level government officials to HRH issues

The HSAs did not explicitly examine high-level government officials’ overall commitment to HRH.

### Voice and participation

This study examined mechanisms used by providers to gain feedback from patients and communities.

#### Patient and community feedback

In Kenya, St. Lucia, and Uganda, the most commonly cited way to gain feedback from patients and the larger community is through suggestion boxes. Community groups also mobilize to provide input on care, particularly related to disease areas with high NGO involvement, such as HIV. Certain provinces in Vietnam have a more institutionalized process to gain community feedback whereby meetings with hospital managers, heads of wards, heads of departments, and patients take place weekly or monthly so that issues can be identified and addressed.

## Discussion

Viewing the health workforce through a governance lens of overall strategic planning capacity, accountability, transparency, information systems, efficiency, equity and fairness, responsiveness, and citizen voice and participation provides a framework for assessing how to strengthen the health workforce holistically and sustainably. While the eight principles presented here are by no means comprehensive of all the many complex ways that health governance impacts the health workforce, they serve to further the conversation on the types of activities that should be researched for health systems strengthening.

We recognize several limitations to this study. First and foremost, the HSA approach was not developed to assess how health governance is operationalized to improve health workforce performance. As a result, additional indicators that could inform this topic were not collected and therefore leave many issues related to HRH and governance unanswered here. Second, not all HSA reports provide comparable information for each indicator because implementation of the HSA approach varied slightly by each assessment team. This was particularly true for the indicator on commitment of high-level government officials to HRH issues, which teams did not routinely assess. Third, given that the HSAs reviewed for this study were completed as long ago as 2007, they do not reflect more recent HRH governance activities. Last, the HSA countries were selected based on the demand of USAID missions, not on a set of more scientific criteria. Despite these limitations, we believe that the HSA findings provide valuable insight into the main strengths of, and challenges to, governance and the health workforce, which are summarized below.

### Main governance strengths

The study identified country-specific interventions that improve governance of the health workforce in many countries. Four strengths are most common. First, routine resource tracking methodologies like NHA are increasing the transparency of financing of the health workforce and broader health system, and countries have been moving towards institutionalization of the methodology so that they are less dependent on external assistance. Kenya and Tanzania have been particularly successful at increasing in country capacity to produce routine NHA estimations, as each country has multiple NHAs.

Second, many countries have been training new cadres of health workers to address critical HRH shortages and ensure that the health workforce is responsive to population health needs. The HSAs for Angola, Kenya, Lesotho, and Uganda discuss how community health workers have become a means to increase access to primary health care.

Third, registration and licensure of health workers is a common practice in most of the countries examined. While few countries have the capacity to re-register or update licensure of health workers, most ensure that certain qualifications are met before medical professionals can begin practicing. Having this initial structure in place to register and license health workers is a foundation from which to expand in the future.

Finally, many countries, including Angola, Kenya, Lesotho, and Mozambique are experimenting with some type of financial or non-financial incentive to increase health worker efficiency and improve health outcomes of patients. As countries implement incentive programs, it is imperative that results are monitored and lessons learned are disseminated to other countries.

### Main governance challenges

Three key challenges were identified in countries examined. First, although many countries recognized the importance of strategic plans, a significant number of countries were still struggling to develop HRH-focused plans and then implement them. Given the financial and human resource investment required for the implementation, it is no surprise that the countries have experienced difficulty moving from plan to action.

Second, overcoming the financial and human resource shortages to improve licensure, regulation, and supervision is a major challenge. The rise of private providers in LMIC has added a new dimension to the quality assurance process that will require adaptation of regulations and systems in countries that have previously focused entirely on public sector systems. In resource-limited settings, resources are easily redirected to direct service delivery expenses. However, the importance of ensuring quality of the workforce, and therefore of services delivered, should not be undervalued.

Third, moving from paper-based to automated health information systems is a challenge. Existing country information systems are limited in their ability to provide timely and relevant information to policymakers, providers, and patients. Common obstacles to implementation include lack of a coordinating body to manage the information systems, lack of an up-to-date health information strategic plan, and lack of standard definitions for data elements collected; having multiple parallel information systems for NGO-led interventions; and having only limited infrastructure for information communication technology and limited demand for health data.

### Possible strategies to improve governance of the health workforce

Several documents exist to guide best practices for developing HRH strategic plans [35,36]. Key messages from these documents include: the importance of creating a steering committee to oversee the development of the strategic plan; the importance of using evidence to inform the plan; and the importance of bringing a wide range of stakeholders to the table to ensure that the plan is relevant to the public, NGO, and private sectors. To ensure that countries are equipped with approaches that work, more research is needed on the specific challenges to implementing a strategic plan and solutions that have been applied to confront these challenges. Furthermore, systems to monitor implementation and evaluate impact of HRH strategies must be developed. For a monitoring and evaluation plan to be effective, it needs to be built into the strategic plan from the beginning, and incorporate indicators that can be easily tracked and are relevant to plan objectives. A recent study by Altman et al. applies a standardized set of 60 indicators in five case countries and presents information concerning the availability, relevance, and ability of indicators that track health system performance [37]. Building off of lessons learned from existing country experiences along with a country’s own specific evaluation can inform the types of indicators to be included in the monitoring and evaluation plan. However, further research into how to effectively integrate monitoring and evaluation into strategic planning for HRH is needed.

To ensure that limited resources devoted to licensure, regulation, and supervision are used efficiently, countries can learn from existing interventions. For example, mentorship programs in Angola consider using foreign doctors to improve supervision of newly trained health workers and some countries are make progress in standardizing their supervisory visits through the use of checklists. Efforts documented in the literature, such as the use of smart phone technology for improving the supervision of tuberculosis patients, can help to provide more regular and consistent follow up with health workers [38]. These types of interventions should be explored in more detail to increase health worker accountability.

As countries build their health information systems, there is a need to ensure that overlapping systems are not produced. The goal of an information system should be to collect relevant and timely information in a manner that requires minimal effort from data reporters and data analysts. Data demands of the national government, district governments, development partners, and civil society need to first be considered. Next, when developing a standardized set of comprehensive indicators, countries need to be cognizant that the effort required to collect data is relative to their ability to inform policy and planning and that the data collection method is feasible. Where possible, existing information systems should be harmonized, and any new system should be flexible so that it can adapt to the dynamic nature of the health system.

Finally, this paper presents multiple examples of innovative country examples that improve governance of the health workforce: in Uganda, the government uses criteria such as the level of care provided and the number of health care providers in a region to determine health worker salaries; in Lesotho, the government is providing health services through a PPP; in Ukraine, in-service training is incentivized through a points-based system; and in Vietnam, community feedback is gained through regular and structured meetings with facility leaders. Further research is required to evaluate the impact of country-specific interventions, and to determine how they can be scaled up within their own country, or used in other countries to improve governance of the health workforce.

## Conclusions

Research into the HRH field, in particular its link to health governance, and in LMIC, is limited, but is expected to grow as initiatives such as the Global Health Workforce Alliance and Frontline Health Workers Coalition spotlight health workforce issues. Priority research questions such as those identified by Ranson et al. [2] provide a framework for researchers and program implementers to use. However, researchers should be careful to approach HRH through a health system strengthening lens, rather than as separate support initiatives. As we found in the HSAs of 20 countries, the breadth of HRH challenges requires strengthening health governance as well, in order to effect change in the health system.

## Endnotes

^a^WHO building blocks include leadership and governance; financing; service delivery; health workforce; medical products, vaccines and technologies and information.

^b^All HSA reports used in this analysis can be found at the Health Systems 20/20 Website: http://www.healthsystems2020.org/content/resource/detail/528/

## Abbreviations

HAF: Human resources for health Action Framework; HRH: Human resources for health; HSA: Health System Assessment; LMIC: Low- and middle-Income countries; MOH: Ministry of Health; NGO: Non-governmental organization; NHA: National Health Accounts; NHIF: National Health Insurance Fund; PPPs: Public-private partnerships; SHIB: Social Health Insurance Benefits; WHO: World Health Organization.

## Competing interests

The authors declare that they have no competing interests.

## Authors’ contributions

JP conceived of the study, and JP and EQ participated in its design and review. SD performed the qualitative data analysis and human resource management literature review. AO performed the governance literature review, and SD and AO drafted the manuscript. All authors read and approved the final manuscript. The authors thank Linda Moll from Abt Associates.
